# COVID-19 and cognitive performance: a Mendelian randomization study

**DOI:** 10.3389/fpubh.2023.1185957

**Published:** 2023-08-22

**Authors:** Ching-Man Tang, Gloria Hoi-Yee Li, Ching-Lung Cheung

**Affiliations:** ^1^Department of Health Technology and Informatics, The Hong Kong Polytechnic University, Hung Hom, Hong Kong SAR, China; ^2^Department of Pharmacology and Pharmacy, The University of Hong Kong, Pokfulam, Hong Kong SAR, China

**Keywords:** SARS-CoV-2 infection, COVID-19, cognitive performance, inflammatory markers, Mendelian randomization

## Abstract

**Background:**

A substantial proportion of individuals with COVID-19 experienced cognitive impairment after resolution of SARS-CoV-2 infection. We aimed to evaluate whether genetic liability to SARS-CoV-2 infection *per se*, or more severe COVID-19, is causally linked to cognitive deficit.

**Methods:**

We firstly performed univariable Mendelian randomization (MR) analysis to examine whether genetic liability to SARS-CoV-2 infection, hospitalized and severe COVID-19 is causally associated with cognitive performance. To dissect the causal pathway, multivariable MR (MVMR) analysis was conducted by adjusting for five inflammatory markers [C-reactive protein, interleukin (IL)-1β, IL-6, IL-8, and tumour necrosis factor α, as proxies of systemic inflammation].

**Results:**

In univariable MR analysis, host genetic liability to SARS-CoV-2 infection was associated with lower cognitive performance [inverse variance weighted (IVW) analysis, estimate: −0.023; 95% Confidence Interval (CI): −0.038 to −0.009]. Such causal association was attenuated in MVMR analysis when we adjusted for the five correlated inflammatory markers in one analysis (IVW analysis, estimate: −0.022; 95% CI: −0.049 to 0.004). There was insufficient evidence of association for genetic liability to hospitalized and severe COVID-19 with cognitive performance.

**Conclusion:**

The causal effect of host genetic liability to SARS-CoV-2 infection on reduced cognitive performance may be mediated by systemic inflammation. Future studies examining whether anti-inflammatory agents could alleviate cognitive impairment in SARS-CoV-2-infected individuals are warranted.

## 1. Introduction

There is emerging evidence that the symptoms of coronavirus disease 2019 (COVID-19), such as cognitive dysfunction, fatigue, and dyspnoea, might persist beyond the acute infection of severe acute respiratory syndrome coronavirus 2 (SARS-CoV-2), which is known as post COVID-19 condition, or long COVID (1). The World Health Organization (WHO) standardized the clinical case definition of post COVID-19 condition as a condition occurring in people with probable or confirmed SARS-CoV-2 infection at ≥3 months from onset of COVID-19 and last for ≥2 months, which are not due to other known diseases (2). Cognitive dysfunction is among the most common long COVID symptoms in WHO’s clinical case definition (2). Typical symptoms include memory and concentration issues (3). Notably, cognitive impairment has impact on the daily function of individuals and significantly lowers their quality of life (4). It is essential to evaluate whether COVID-19 would causally lead to cognitive impairment in the long-term, to facilitate health management of people who have ever been infected.

Cross-sectional (5) and cohort studies (6, 7) revealed that more than 50% of SARS-CoV-2-infected individuals experienced cognitive impairment after they had recovered from the infection. However, as cognitive performance of the study participants prior to SARS-CoV-2 infection were often unmeasured, causal inference cannot be resolved in these studies. Meanwhile, SARS-CoV-2 infection and COVID-19 hospitalization were found to have inverse genetic correlation with cognitive performance (8), and our previous Mendelian randomization (MR) study showed that from the genetic perspective, lower cognitive intelligence (measured by neurocognitive tests evaluating the fluid domain of cognitive performance, including reasoning, verbal ability and memory) was associated with elevated risk of COVID-19 hospitalization (9). It is unknown if the inverse causal relationship of genetically susceptibility to COVID-19 clinical phenotypes with cognitive performance also contribute to the high prevalence of cognitive impairment among SARS-CoV-2-infected individuals. Although a meta-analysis recently revealed a cognitive decline in people infected with SARS-CoV-2 (10), the heterogeneous populations of the constituting studies ranged from community-dwelling individuals to hospitalized patients with different COVID-19 severity levels. It remains unclear if people with SARS-CoV-2 infection *per se*, or people reaching certain levels of COVID-19 severity, would have cognitive complaints. Moreover, current observational studies were limited by short follow-up period. Even if COVID-19 was shown to cause cognitive deficit, the duration of effects remains unknown.

By adopting the two-sample MR approach, we aimed to evaluate the causal effect of genetic liability to three COVID-19 clinical phenotypes (SARS-CoV-2 infection, COVID-19 hospitalization, and COVID-19 severity) on cognitive performance. In case such causal association exists, we additionally aimed to identify the potential mediators in the causal pathway.

## 2. Materials and methods

### 2.1. Study design

**Figure 1:**
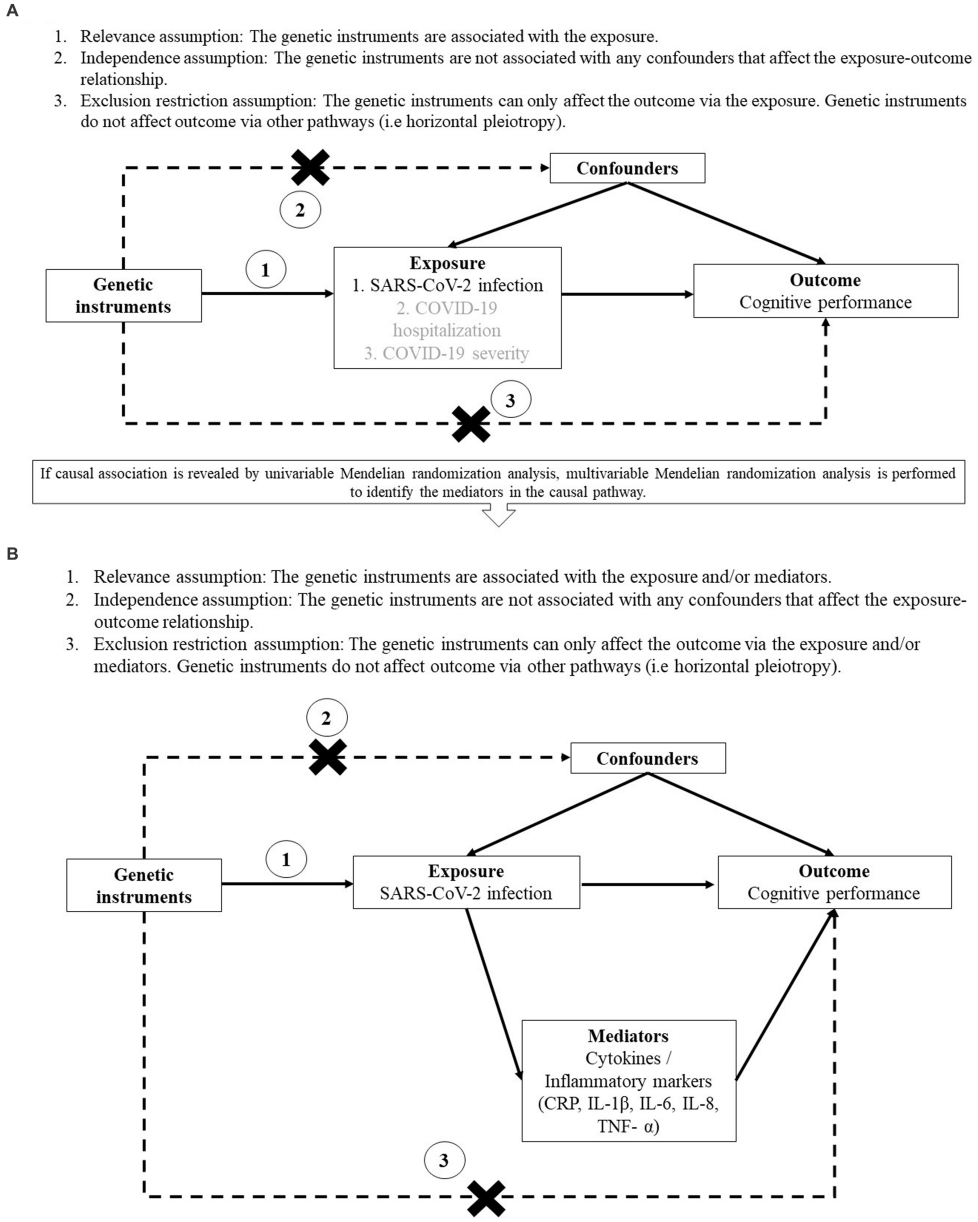
Study design and assumptions of univariable **(A)** and multivariable **(B)** Mendelian randomization analysis. **(A)** Assumptions of univariable Mendelian randomization study. **(B)** Assumptions of multivariable Mendelian randomization study.

The study design and assumptions of MR are illustrated in Figure 1. The MR approach utilizes genetic variants as instrumental variables for the exposure, making it feasible to examine the lifelong and causal effects of the exposure on the outcome (11). It resembles randomized controlled trails in which alleles are randomly allocated at conception, such that it is less susceptible to reverse causation and unmeasured confounding when compared to conventional cohort studies, thus providing stronger evidence of causal inference. In particular, our current study utilizes the two-sample MR approach by obtaining the summary statistics of the exposure and outcome from two independent samples available in the public domain (12). Despite the advantages of two-sample MR approach, the efficiency and robustness of the findings depend on three assumptions: (a) The genetic instruments are associated with the exposure; (b) The genetic instruments are not associated with any confounders that affect the exposure-outcome relationship; (c) The genetic instruments can only affect the outcome via the exposure but not via other pathways (11).

In the major analysis, we used univariable MR approach to evaluate if genetic liability to COVID-19 clinical phenotypes was causally associated with cognitive performance. If any causal relationship was identified from the major analysis, we employed multivariable MR (MVMR) approach to examine whether the potential mediators played a role in the causal pathway. These potential mediators were proxies of systemic inflammation including blood levels of C-reactive protein (CRP) and cytokines [interleukin-1β (IL-1β), interleukin-6 (IL-6), interleukin-8 (IL-8), tumour necrosis factor α (TNF-α)], since systemic inflammation was suggested to play a role in cognitive dysfunction among SARS-CoV-2-infected individuals (13, 14). MVMR was conducted by adjusting for the beta estimate of each potential mediator separately and adjusting for all the potential mediators in one single analysis due to their correlation with each other.

### 2.2. Data source

Publicly available summary statistics were retrieved from the genome-wide association studies (GWAS) conducted in Europeans. Release 5 of the GWAS meta-analysis of the three COVID-19 clinical phenotypes performed by the COVID-19 host genetics initiative (HGI) (15) were chosen to be the data source instead of Release 7, as Release 5 is currently the latest one with available summary statistics from Europeans upon exclusion of the UK Biobank participants. This was to prevent sample overlap with the outcome datasets that also comprised UK Biobank participants, since such overlap may lead to bias (16). Cognitive performance was the outcome of interest, with summary statistics extracted from a GWAS meta-analysis, at which the covariance between various domains of cognitive function were captured by the general intelligence or Spearman’s *g* (17). Details of data source for the exposure (15), outcome (17) and mediators (18–20), including their participant selection criteria, assessment methods, ancestry, and sample size, are described in Table 1.

**Table 1:** Data source in the current Mendelian randomization study.

Traits	Description	Ancestry	Sample size
COVID-19 clinical phenotypes (exposure)			
SARS-CoV-2 infection (15)	A meta-analysis of GWAS with case–control study design. Cases included individuals with SARS-CoV-2 infection identified by laboratory confirmation, electronic health record, clinical investigation or self-reported, regardless of the presence of symptoms. Controls were individuals who did not have known SARS-CoV-2 infection.	100% European, participants from UK Biobank were excluded.	Total: 1,348,701 Cases: 32,494 Controls: 1,316,207
COVID-19 hospitalization (15)	A meta-analysis of GWAS with case–control study design. Cases included individuals who were hospitalized due to symptoms associated with COVID-19, with laboratory-confirmed SARS-CoV-2 infection. Controls were people who did not have known SARS-CoV-2 infection.	100% European, participants from UK Biobank were excluded.	Total: 1,557,411 Cases: 8,316 Controls: 1,549,095
COVID-19 severity (15)	A meta-analysis of GWAS with case–control study design. Cases were critically ill patients with laboratory-confirmed SARS-CoV-2 infection, who required respiratory support or died from COVID-19-associated symptoms. Controls were those who did not have known SARS-CoV-2 infection.	100% European, participants from UK Biobank were excluded.	Total: 1,059,456 Cases: 4,792 Controls:1,054,664
Cognitive trait (outcome)			
Cognitive performance (17)	A GWAS meta-analysis comprising 14 cohorts of cognitive performance measured by different neurocognitive tests evaluating the fluid domain of cognitive performance, including reasoning, verbal ability and memory. Although different cohorts adopted different neurocognitive tests in assessing the cognitive performance of study participants, a common latent g factor underlying various cognitive domains was investigated. Study participants with cognitive decline were excluded.	100% European	269,867
Inflammatory markers (mediators in multivariable Mendelian randomization analysis)			
CRP (19)	A GWAS meta-analysis including participant from UK Biobank and CHARGE consortium. Serum CRP levels were measured by immunoturbidimetry assay (Beckman Coulter AU5800) and were natural log transformed. Individuals with extreme CRP level, auto-immune diseases and use of immune-modulating drugs were excluded.	100% European	575,531
IL-6 (20)	A GWAS meta-analysis comprising 13 cohorts. Proteins were measured by immunoassay (proximity extension assay). In each constituting cohort, inverse-normal transformation and/or standardization were applied to the normalized protein expression levels.	100% European	21,758
IL-8 (20)			
IL-1β (18)	A GWAS meta-analysis comprising three studies conducted in Finnish population. IL-1β and TNF-α were among the 41 serum or plasma cytokines tested. The cytokine levels were measured by enzyme-linked immunoassay and normalized by inverse transformation, with effect size presented in standard deviation.	100% European	3,309
TNF-α (18)			3,454

CRP, C-reactive protein; IL-6, interleukin 6; IL-8, interleukin 8; IL-1β, interleukin 1β; TNF-α, tumour necrosis factor α; CHARGE consortium, The Cohorts for Heart and Aging Research in Genomic Epidemiology (CHARGE) consortium.

### 2.3. Selection of genetic instruments

The details of identifying independent single nucleotide polymorphisms (SNPs) and selecting initial genetic instruments for the three COVID-19 clinical phenotypes were described in our previous study (21). Briefly, for each of the three phenotypes, clumping was performed on all the genome-wide significant SNPs by PLINK 1.9 (22), using *r*^2^ threshold of 0.01 and 10-Mb window with reference to the European reference panel of the 1,000 Genomes Project. In the primary analysis, due to the few independent SNPs reaching genome-wide significance threshold (*p* < 5×10^−8^), a suggestive significance threshold (*p* < 5×10^−6^) was firstly adopted to select the initial genetic instruments for the COVID-19 clinical phenotypes. If significant association was found, a sensitivity analysis using genome-wide significant instruments was performed. For palindromic instruments, or instruments that are absent from the mediator (for MVMR only) and/or outcome datasets, proxies in high linkage disequilibrium (LD) (*r*^2^ ≥ 0.8) with the initial instruments and significantly associated with the exposure were identified. To avoid bias due to reverse causation, genetic instruments that explained more on the outcome (cognitive performance) than the exposure (COVID-19 clinical phenotypes) were excluded by MR Steiger filtering (23). We also checked if the independence and exclusion restriction assumptions were violated (Figure 1). We examined if the instruments have genome-wide significant association with potential confounders [such as education attainment (8, 24) and body mass index] (25, 26) or alternative risk factors that affect cognitive performance (but not via SARS-CoV-2 infection or COVID-19 severity) in large-scale representative GWAS/GWAS meta-analysis using PhenoScanner (27). The effect alleles of the instruments were oriented such that they had a positive association with the exposure (i.e., SARS-CoV-2 infection).

### 2.4. Statistical analysis

Inverse variance weighted (IVW) method, assuming all instruments are valid (28), is the main MR analysis. Since IVW method might be prone to bias in the presence of invalid instruments, we used weighted median, contamination mixture, MR-Egger and MR pleiotropy residual sum and outlier (MR-PRESSO) methods as the sensitivity analysis. The weighted median method could produce a consistent estimate even when up to 50% of the instruments are invalid (29). Contamination mixture method makes the plurality valid assumption, having reasonable power and the lowest mean squared error with reference to other MR methods (30). The MR-Egger intercept indicates the average pleiotropic effects of the genetic instruments, while the slope coefficient takes into account the presence of unbalanced directional pleiotropy and provides a valid causal effect estimate (31), despite the relatively low power (32). MR-PRESSO global test detects the presence of pleiotropic outliers and the outlier test corrects the causal estimate upon removal of outliers (33). In the current study, horizontal pleiotropy was detected by MR-Egger intercept and MR-PRESSO global tests. Pleiotropic outliers identified by MR-PRESSO global test, if any, were excluded, followed by the repetition of the main IVW and other sensitivity analyses. In addition, Cochran’s Q test was performed to assess the heterogeneities across the instruments.

Compared to univariable MR analysis which evaluates the total effect of the exposure on the outcome, MVMR approach examines the direct effect of the exposure on the outcome by keeping the potential mediators constant (34, 35). Hence, several MVMR methods that built on common univariable MR approaches were utilized in the current study to understand the potential causal mechanisms if significant association was revealed in univariable analysis. These MVMR methods include MVMR-IVW (28), MVMR-Median (36), MVMR-Egger (37), MVMR-PRESSO and MVMR-Robust (33). MVMR-Robust is an extension of MVMR-IVW by utilizing the MM-estimation as the robust regression method (36). The MVMR-PRESSO global test (33) and MVMR-Egger intercept test (37) were adopted to detect residual pleiotropy. While these MVMR methods were developed based on different assumptions, each of these methods has certain advantages under some scenarios and they were all included as sensitivity analysis in MVMR (36). Having robust results across these sensitivity analyses would strengthen the confidence of the finding. As the small number of instruments reaching genome-wide significance is insufficient for some MVMR analysis, only instruments suggestively associated with the COVID-19 clinical phenotypes were adopted in MVMR approach.

This MR study examined the causal association of binary exposures (COVID-19 clinical phenotypes) with continuous outcomes (cognitive performance). To facilitate interpretation, the causal estimates were presented as change in cognitive performance [in standard deviation (SD)] per doubling the prevalence of COVID-19 clinical phenotypes, by multiplying the original beta estimate (per log odds change in the exposure) by 0.693 (38). Bonferroni correction was applied to account for multiple testing when we assess the casual association of genetic liability to three COVID-19 clinical phenotypes with cognitive performance (0.05/3 = 0.017). All MR analyses were conducted using R (version 4.1.3) with the “MendelianRandomization,” “TwoSampleMR,” “MRPRESSO” and “MVMR” packages. For each MR analysis, the statistical power was calculated using a web-based calculator^1^ (39). Plots of power against the causal estimate of outcome are presented in Supplementary Figures S1–S3.

## 3. Results

### 3.1. Univariable MR analysis

**Figure 2:**
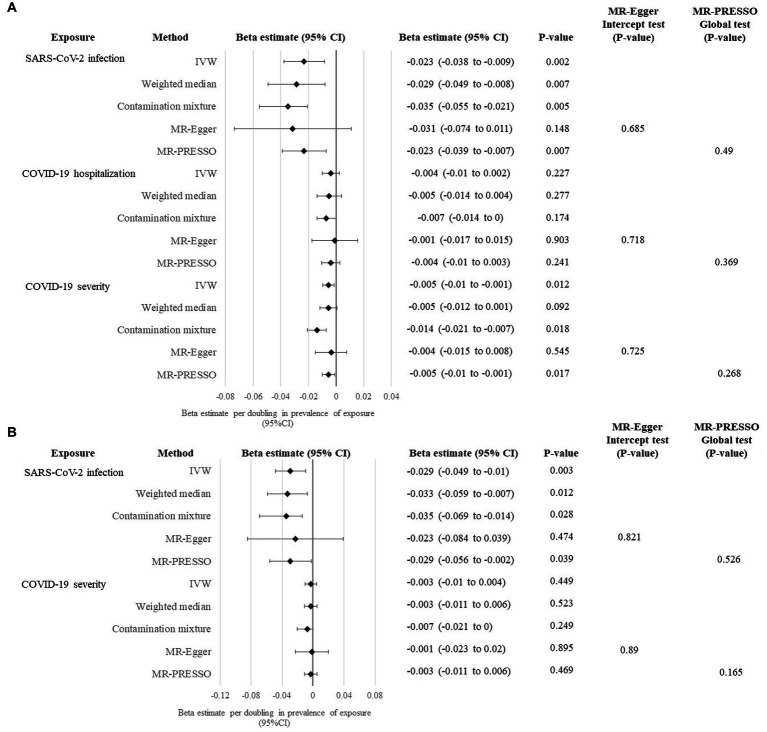
Univariable MR analysis evaluating causal effects of genetic liability to COVID-19 clinical phenotypes on cognitive performance. **(A)** Primary analysis evaluating the causal effects of genetic liability to SARS-CoV-2 infection, COVID-19 hospitalization and COVID-19 severity with cognitive performance using instruments with suggestive significance at *p* < 5×10^−6^. **(B)** Sensitivity analysis evaluating the causal effects of genetic liability to SARS-CoV-2 infection and COVID-19 severity with cognitive performance using genome-wide significant instruments at *p* < 5×10^−8^.

A summary of the genetic instruments adopted in the univariable MR analysis [number of instruments included in the analysis, proportion of variance explained on the exposure, F statistics (as a measure of the strength of instruments), and Cochrane’s Q statistics] are listed in Table 2. We firstly performed the primary univariable analysis using instruments with suggestive association with the exposure. In the main IVW analysis, significant association was observed for genetic liability to SARS-CoV-2 infection with lower cognitive performance after correction for multiple testing [per doubling in prevalence of SARS-CoV-2 infection, estimate: −0.023 SD, 95% confidence interval (CI): −0.038 to −0.009, *p*: 0.002; Figure 2A]. We found similar findings in sensitivity analyses of weighted median, contamination mixture, and MR-PRESSO methods, but a wider CI crossing the null was observed for the MR-Egger regression method (Figure 2A). We could not observe any causal relationship of genetic susceptibility to COVID-19 hospitalization with cognitive performance (Figure 2A). Genetic susceptibility to severe COVID-19 was associated with poorer cognitive performance in IVW analysis (per doubling in the prevalence of severe COVID-19, estimate: −0.005, 95% CI: −0.01 to −0.001, *p*: 0.012), contamination mixture and MR-PRESSO methods (Figure 2A).

**Table 2:** Summary of genetic instruments included in the univariable MR analysis.

Exposure	Outcome	Number of genetic instruments included in the analysis^a^	Variance explained by the genetic instruments on exposure (%)	F statistics	Cochran’s Q Test	
					Q-statistic	Heterogeneity *p*-value
SARS-CoV-2 Infection	Cognitive performance	16 (19-2-0-0-1^b^)	0.55	457.73	15.161	0.44
		Sensitivity analysis: 5 (5-0-0-0-0)	Sensitivity analysis: 0.29	Sensitivity analysis: 777.58	Sensitivity analysis: 3.77	Sensitivity analysis: 0.438
COVID-19 Hospitalization		22 (28-5-1-0-0)	3.27	2,393.1	22.887	0.35
COVID-19 Severity		34 (40-5-1-0-0)	7.58	2,555.6	37.767	0.261
		Sensitivity analysis: 10 (10-0-0-0-0)	Sensitivity analysis: 3.4	Sensitivity analysis: 3,723.87	Sensitivity analysis: 13.698	Sensitivity analysis: 0.134

a Number of genetic instruments included in the analysis = Number of independent genetic instruments significantly associated with the exposure (revealed from the GWAS meta-analysis of exposure) − number of genetic instruments that cannot be matched with the outcome but no proxies can be identified − number of genetic instruments removed by Steiger filtering − number of outliers identified by MR-PRESSO global test − number of genetic instruments that violated the independence and/or exclusion restriction assumption.

b One genetic instrument (rs1853415) was excluded from the analysis as it was associated with education attainment (65) (*p* = 9.62 × 10^−9^), which is a confounder of the exposure-outcome association.

In the sensitivity analysis using genome-wide significant instruments, the association of genetic liability to SARS-CoV-2 infection with cognitive performance yielded similar causal estimates in the main IVW analysis (estimate: −0.029, 95% CI: −0.049 to −0.01, *p*: 0.003) and other sensitivity analyses, except for MR-Egger regression method (Figure 2B). Conversely, causal association could not be seen in all univariable MR methods for genetic susceptibility to severe COVID-19 on cognitive performance when genome-wide significant instruments were employed (Figure 2).

### 3.2. MVMR analysis

**Figure 3:**
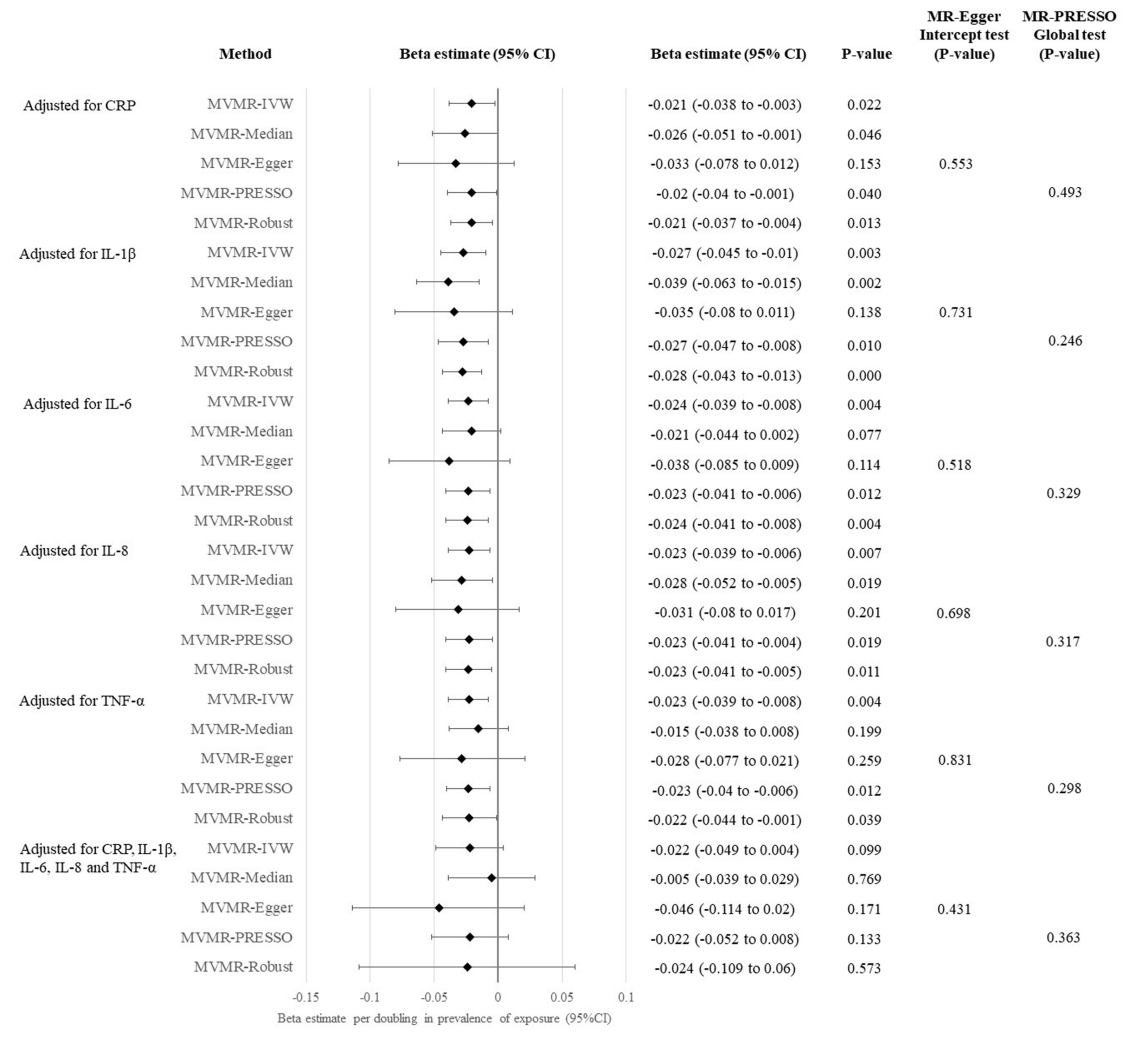
Multivariable MR analysis evaluating causal effects of genetic liability to SARS-CoV-2 infection on cognitive performance.

Since both the primary and sensitivity analyses revealed a significant causal association for genetic liability to SARS-CoV-2 infection with cognitive performance using univariable MR approach, we performed MVMR analysis to identify the causal mediators. When we adjusted for the genetically determined levels (i.e., Levels of inflammatory markers are estimated by the effect size of the association between the genetic variants and the inflammatory marker obtained from published and publicly available GWAS) of the five inflammatory markers (including CRP, IL-1β, IL-6, IL-8 and TNF-α) in blood separately, the causal association remained significant in at least three of the five tested MVMR methods, while the causal estimate and the CI were driven towards the null (Figure 3). Due to the correlation between the inflammatory markers, we further adjusted for the genetically determined levels of all the five inflammatory markers in one single MVMR analysis. Attenuation of causal association was observed in IVW analysis (estimate: −0.022; 95% CI: −0.049 to 0.004, *p*: 0.099) and other tested MVMR methods (Figure 3).

In the univariable and multivariable MR analyses conducted in this study, all the MR-Egger intercept and MR-PRESSO global tests were statistically insignificant (*p* > 0.05).

## 4. Discussion

This two-sample MR study revealed that host genetic liability to SARS-CoV-2 infection was causally associated with lower cognitive performance. MVMR analysis suggested that genetic susceptibility to systemic inflammation may mediate the causal pathway. There was insufficient evidence to support the causal association of host genetic susceptibility to COVID-19 hospitalization and severity with cognitive performance.

Our MR study demonstrated that genetic liability to SARS-CoV-2 infection was causally associated with impaired cognitive performance. Similar causal estimates were observed using various MR methods in the primary analysis utilizing genetic instruments suggestively associated with SARS-CoV-2 infection, and the sensitivity analysis using genome-wide significant instruments. Such robust finding is partially consistent with a number of published observational studies (5, 7, 40–46). Among these studies, the prospective cohort study with the largest sample size (1,284,237 SARS-CoV-2-infected individuals and controls respectively) and longest follow-up period (2 year) to-date demonstrated that the risk of cognitive deficit was still increasing by the end of follow-up, with a hazard ratio of 1.36 when compared to individuals with other respiratory tract infection (42). Longer follow-up of the individuals with SARS-CoV-2 infection will be required to examine if the infection may exert a long-term effect on the cognitive function, as suggested by the current study findings that genetic liability to SARS-CoV-2 infection may pose a lifelong risk of cognitive deficit. A recently published MR study reported that genetic susceptibility to hospitalized and critical COVID-19, but not SARS-CoV-2 infection, was associated with an elevated risk of Alzheimer’s disease (AD) (47). While the onset of AD mainly occurs at the age of mid-60s, the GWAS of cognitive performance examined by our study included participants with an extensive age range from 5 to 98 (17). Our study added to the current evidence that genetic liability to SARS-CoV-2 infection *per se* (irrespective of disease severity) could lead to cognitive deficit, which is applicable to individuals of all ages. Contradictory findings were observed for the cognitive ability among the young individuals with and without SARS-CoV-2 infection (48, 49). It was likely attributed to their small sample size in the total sample (*n* ~ 200) and the sub-group analysis. Whether SARS-CoV-2 infection has differential effects on the cognitive performance in individuals of different age-groups require future investigation.

One common hypothesized mechanism underlying cognitive impairment in SARS-CoV-2 infected individuals is the indirect damage of brain tissue through systemic inflammation (50). Our MVMR analysis showed that the causal association of genetic liability to SARS-CoV-2 infection with cognitive performance was attenuated after adjustment for genetically determined levels of CRP, IL-1β, IL-6, IL-8 and TNF-α in one single analysis, suggesting that genetic susceptibility to systemic inflammation (proxied by the genetically determined levels of inflammatory markers) might mediate the causal pathway. When compared to healthy individuals, SARS-CoV-2 infected individuals had significantly higher blood levels of several pro-inflammatory cytokines, including IL-1β (51), IL-6 (51, 52), IL-8 (51–53) and TNF-α (51–53). Among these cytokines, levels of IL-1β, IL-6 and TNF-α were also significantly elevated in individuals with ongoing post-acute sequelae of COVID-19, and they were positively correlated with each other (54). Meanwhile, cytokines such as IL-6 and TNF-α stimulate CRP production by liver (55), so increased CRP level was also observed in individuals with SARS-CoV-2 infection (56). Increased levels of pro-inflammatory cytokines and the inflammatory marker CRP were linked to systemic inflammation in COVID-19 patients. Systemic inflammation level at acute infection was found to predict neurocognitive performance (14). Systemic inflammation might disrupt the blood–brain barrier, enabling cytokines to enter the central nervous system, where the cytokines activate the microglia and cause myelin loss in the brain (13, 57), contributing to cognitive impairment. Notably, none of the five tested inflammatory markers can individually attenuate the causal association of genetic liability to SARS-CoV-2 infection with cognitive impairment in MVMR, implying that the correlated inflammatory markers work together to mediate the causal pathway. Nevertheless, we could not exclude the possibility that the small sample-size of the mediator datasets may limit the power of the MVMR analysis adjusted for individual inflammatory marker, which should be re-visited when larger GWAS dataset becomes available. Direct invasion of SARS-CoV-2 into the central nervous system is another commonly hypothesized mechanism that may play a role in the causal pathway from SARS-CoV-2 infection to cognitive deficit (50). However, MR study design could not examine this mechanism and further investigations are warranted.

Insufficient evidence could support a causal association of genetic susceptibility to COVID-19 hospitalization and severity with cognitive function. Our findings are in line with most cohort studies reporting that post COVID-19 cognitive impairment is independent of COVID-19 severity (5, 6, 43, 58–60). One possible reason may be due to the different host genetics that affect the liability to SARS-CoV-2 infection and progression to COVID-19 hospitalization/severity, as explained in our previous MR study (19). Even though genetic liability to SARS-CoV-2 infection was casually linked to a higher risk of cognitive deficit, the same causal effect might not necessarily exist for those who are genetically susceptible to COVID-19 hospitalization/severity. In addition to cognitive impairment, the prevalence of several long COVID symptoms, including fatigue, headache, anosmia, depression and physical performance, are comparable among SARS-CoV-2 infected individuals with various levels of disease severity (61, 62). It was hypothesized that the presence of long COVID symptoms might be related to persistent systemic inflammation rather than disease severity, as more than 22% of the study participants had elevated CRP level even at 5 months after hospital discharge (61). Nonetheless, several studies demonstrated that cognitive deficit in SARS-CoV-2 infected individuals were correlated with the severity of the COVID-19 symptoms (40, 63, 64). Differences in study design, study population, use of covariates, and time of assessment might explain the discrepancy.

This study has clinical implications. Our study provides evidence that genetic liability to SARS-CoV-2 infection *per se*, regardless of COVID-19 severity, had an adverse causal effect on cognitive performance, which applies to individuals of all ages. Cognitive deficit not only affects the overall wellbeing of an individual, but also increases the burden brought to the caregivers and the healthcare system. Implementation of appropriate screening and rehabilitation programmes in relation to cognitive performance is an essential part of the management strategy of SARS-CoV-2 infected individuals, as a substantial proportion of the global population may have contracted the virus. More importantly, our study demonstrated from the perspective of genetic susceptibility that systemic inflammation might mediate the causal pathway from SARS-CoV-2 infection to cognitive impairment. Such finding led to the hypothesis that anti-inflammatory agents might help to alleviate the cognitive deficit problems among SARS-CoV-2 infected individuals. While current clinical trials mainly aim to evaluate whether the blockade of cytokines might improve the survival of SARS-CoV-2 infected individuals, further studies are warranted to examine if blockade of cytokines might alleviate the cognitive impairment problems of the infected individuals.

This study has several strengths. First, we utilized the MR approach to assess the causal association of genetic susceptibility to COVID-19 clinical phenotypes with cognitive performance, which is infeasible in observational studies and experimental trials. Second, we have conducted the MR Steiger test of directionality, ensuring that the genetic instruments for the COVID-19 clinical phenotypes were not subjected to reverse causality. Third, this study had high statistical power to detect a small effect size (Supplementary Figures S1–S3). Fourth, the high F statistic of the genetic instruments (Table 2) indicated weak instrument bias is not likely. Fifth, the findings of this study were robust as confirmed by multiple sensitivity analyses. Nonetheless, there are limitations. First, MR studies rely on a key assumption that the genetic instruments only act on the outcome via the exposure. Although this study yielded insignificant MR-Egger intercept and MR-PRESSO global tests, the possibility of horizontal pleiotropy cannot be completely ruled out. Second, while sample overlapping in two-sample MR analysis might lead to bias towards the observed association (16), there is one overlapping cohort (Genes for Good; 0.38% sample overlap) between the GWAS of SARS-CoV-2 infection and cognitive performance. Assume the bias of the observational estimate is 2.518 per SD increase in the exposure [estimated from the largest cohort study to-date at 2-year follow-up (42)], the bias and type I error were 0 and 0.05 respectively, which is minimal. Third, the MR-Egger regression resulted in insignificant association in all the analyses, most likely attributable to its lower statistical power with reference to other MR methods (31, 32), such as weighted median, contamination mixture and MR-PRESSO, which suggested the presence of causal association. Our study findings are unlikely to be false positive. Fourth, inconsistent results were observed among MVMR methods when we adjusted for the genetically determined levels of inflammatory markers separately. One plausible reason is due to the different assumptions made by different MVMR methods built on common univariable MR approaches, resulting in different causal estimates. Another possible explanation is that the power and Type I error rate of various MVMR methods vary under different scenarios with different proportion of invalid instruments, as demonstrated by a simulation study (36). Nevertheless, all the MVMR methods consistently demonstrated attenuation of causal association when we adjusted for all five inflammatory markers in one single analysis, providing robust evidence that genetic susceptibility to systemic inflammation mediated the causal pathway from SARS-CoV-2 infection to cognitive performance. Fifth, the GWAS or GWAS meta-analysis from which the summary statistics were retrieved for this study only included Europeans, the generalizability to other population is unknown. Sixth, MR study design cannot assess whether physiological changes induced by SARS-CoV-2 infection might lead to cognitive impairment. Future investigations are warranted.

In conclusion, this MR study suggested host genetic liability to SARS-CoV-2 infection, regardless of COVID-19 severity, increases the risk of cognitive impairment. Such association may be mediated by genetic susceptibility to systemic inflammation. Cohort studies with long follow-up duration are required to examine if the effect is persistent. Future studies investigating whether anti-inflammatory agents might alleviate the cognitive deficit issue in SARS-CoV-2 infected individuals are warranted.

## Data availability statement

Publicly available datasets were analyzed in this study. This data can be found here: COVID-19 clinical phenotypes (https://www.covid19hg.org/); cognitive performance (https://ctg.cncr.nl/); CRP (GWAS Catalog accession code GCST90029070, https://www.ebi.ac.uk/gwas/studies/GCST90029070); IL-6 and IL-8 (https://zenodo.org/record/2615265#.ZGIWD3ZBw2w); IL-1β (GWAS Catalog accession code GCST004448, https://www.ebi.ac.uk/gwas/studies/GCST004448); TNF-α (GWAS Catalog accession code GCST004426, https://www.ebi.ac.uk/gwas/studies/GCST004426).

## Ethics statement

Ethical review and approval was not required for the study on human participants in accordance with the local legislation and institutional requirements. Written informed consent was not provided because this MR study was conducted using publicly available and anonymized summary statistics only. The GWAS or GWAS meta-analysis from which the summary statistics were retrieved for this study have obtained ethics approval from respective institutional review board and obtained written informed consent from their study participants.

## Author contributions

GH-YL conceptualized the study. GH-YL and C-MT conducted the analysis and drafted the manuscript. C-LC revised the manuscript critically for important intellectual content. All authors contributed to the article and approved the submitted version.

## Conflict of interest

The authors declare that the research was conducted in the absence of any commercial or financial relationships that could be construed as a potential conflict of interest.

## Publisher’s note

All claims expressed in this article are solely those of the authors and do not necessarily represent those of their affiliated organizations, or those of the publisher, the editors and the reviewers. Any product that may be evaluated in this article, or claim that may be made by its manufacturer, is not guaranteed or endorsed by the publisher.

## Footnotes

^1^ https://sb452.shinyapps.io/power/
