# Supplementary material for: COVID-19 and cognitive performance: a Mendelian randomization study
Source: Front Public Health. 2023 Aug 22;11:1185957. doi: 10.3389/fpubh.2023.1185957 (PMC10477606; doi:10.3389/fpubh.2023.1185957)
Supplement: Supplementary file 1 [file Data_Sheet_1.DOCX]

**Table of Contents**

**Supplementary Figure 1.** Power of MR analysis in evaluating the causal association of genetic liability to SARS-CoV-2 infection with cognitive performance

**Supplementary Figure 2.** Power of MR analysis in evaluating the causal association of genetic susceptibility to COVID-19 hospitalization with cognitive performance

**Supplementary Figure 3.** Power of MR analysis in evaluating the causal association of genetic susceptibility to COVID-19 severity with cognitive performance

**Supplementary Figure 1.** Power of MR analysis in evaluating the causal association of genetic liability to SARS-CoV-2 infection with cognitive performance

**Supplementary Figure 2.** Power of MR analysis in evaluating the causal association of genetic susceptibility to COVID-19 hospitalization with cognitive performance

**Supplementary Figure 3.** Power of MR analysis in evaluating the causal association of genetic susceptibility to COVID-19 severity with cognitive performance
